# From Algorithms to Assets: A Comprehensive Review of AI’s Role in Preclinical Drug Discovery and the Hurdles to Clinical Translation

**DOI:** 10.3390/ph19050696

**Published:** 2026-04-28

**Authors:** Mengqi Cai, Tiancai Liu

**Affiliations:** Key Laboratory of Antibody Engineering of Guangdong Higher Education Institutes, School of Laboratory Medicine and Biotechnology, Southern Medical University, Guangzhou 510515, China; caiyu1235@163.com

**Keywords:** AI, drug discovery, machine learning, deep learning, drug–target interaction, de novo drug design, virtual screening

## Abstract

The integration of artificial intelligence (AI) and big data is poised to significantly augment drug research and development, offering the potential to address persistent challenges such as lengthy timelines and high failure rates. This review provides a critical overview of AI applications across the preclinical drug discovery pipeline (the 2020–2026 literature), covering drug–target interaction prediction, structure prediction, de novo design, virtual screening, drug repurposing, and ADMET forecasting. Beyond surveying technical developments, we critically discuss key translational hurdles, including data quality, model interpretability, patient heterogeneity, and regulatory adaptation, and provide structured summaries of representative models. We conclude by outlining future directions, such as multimodal AI, digital twins, and closed-loop automation, that aim to bridge the gap between computational prediction and clinical application. This review aims to inform researchers and accelerate the delivery of safe and effective therapies.

## 1. Introduction

**Figure 1:**
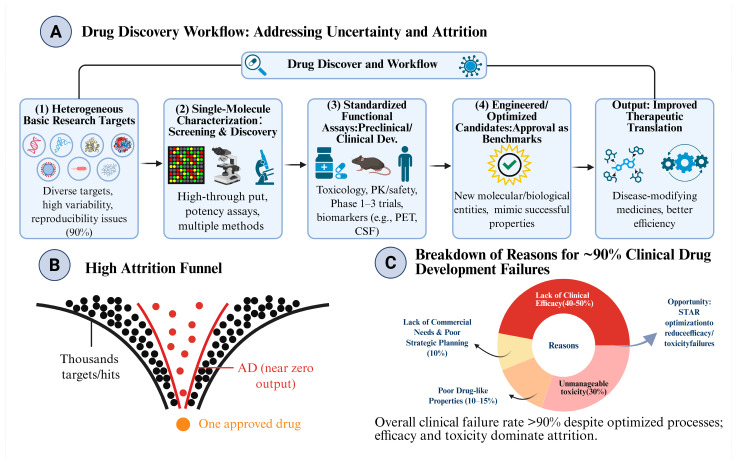
The drug discovery workflow and its clinical attrition landscape. (**A**) Drug Discovery and Development Workflow. The methodical procedure developed to manage biological unpredictability involves heterogeneous foundational research objectives (Step 1), followed by individual molecule evaluation (Step 2), and streamlined functional assays (Step 3), concluding with the production of engineered clinical candidates (Step 4). (**B**) High Attrition Funnel. Here, we can see a dramatic decrease, from thousands of proposed/initial targets/hits to one approved drug (orange). The red curve shows the above-average attrition rate seen in complicated CNS conditions, such as Alzheimer’s Disease (AD), leading to almost no output. (**C**) Breakdown of Clinical Failures. The >90% clinical failure rate is attributable to the following reasons. Analyze first the reasons for clinical inefficiency and unmanageable toxicity. Clinical inefficiency accounts for 40–50% of the failure rate, while unmanageable toxicity accounts for 30% of the clinical failure rate. Both reasons highlight the focus of the need for improvement/ optimization on these issues. For this purpose, example strategies (STAR optimization) need to be considered. (Data source: Duxin Sun et al., 2022 [15]). Created in BioRender. Cai, M. (2026) https://BioRender.com/amd01kh (accessed on 20 April 2026).

Traditional drug discovery methods face several problems, including being extremely costly, lengthy, and high in the rate of unsuccessful attempts. The average cost of drug development is approximately $2.6 billion and takes 10 to 15 years to complete. 90% of clinical trials fail, largely because a new drug is ineffective or extremely toxic [1,2,3]. The complexity of the drug development process contributes to high attrition. This is shown in Figure 1. The issue is further exacerbated by the growing need for precision medicine and treatment for complex diseases [4]. There is a clear need to go beyond the traditional drug research and development methods.

Artificial intelligence (AI), particularly its subfields of machine learning (ML) [5] and deep learning (DL) [6], has become a prominent element in the analysis of biomedicine big data and is a leading factor in redefining the drug discovery process [4,7]. The incorporation of AI into the drug discovery process transforms a traditional, trial-and-error process into a more refined, data- and intelligence-driven process [8]. The incorporation of some specific algorithms for certain critical processes has driven innovations in the field of drug discovery [8]. Graph Neural Networks (GNNs) have been utilized for the modeling of molecular systems [9,10,11,12]; Convolutional Neural Networks (CNNs) predict drug–target interactions from 2D structural representations of compounds [10]; Reinforcement learning (RL) has been used for training generative models via policy gradient [11,12]. Furthermore, Natural Language Processing (NLP) has been utilized for extracting key information from the biomedical literature and constructing knowledge graphs to support drug repurposing and biomarker discovery [12]. Specific architectures, such as graph neural networks, transformers, and diffusion models, have enabled more predictive and generative processes, reducing the time of drug evaluation and discovery while improving efficiency [9,10,11,12].

Despite the growing number of reviews on AI use in drug discovery, from method-focused surveys to pipeline-oriented syntheses incorporating clinical milestones, a work that provides a comprehensive overview of AI architectures across critical tasks, dissects translational hurdles from target to patient, and integrates emerging paradigms such as digital twins remains absent [8,13,14]. Therefore, the major aim of this review is to provide an up-to-date and holistic overview of the role of AI across all key stages of drug discovery, with a particular focus on emerging technologies and their integration into real-world applications (limitations and future directions). To this end, the three objectives are: survey cutting-edge AI models across key drug discovery tasks; analyze limitations impeding clinical translation; outline future directions toward clinical integration.

## 2. Search Strategy and Selection Criteria

This is a narrative review, not a systematic review. To ensure transparency, we followed a structured search and selection process as described below.

### 2.1. Literature Search

We searched PubMed, MEDLINE, Embase, Cochrane Library, Web of Science, and Google Scholar for peer-reviewed articles published between January 2020 and April 2026. Search terms covered three categories:

Category 1 (AI Methodologies): artificial intelligence, machine learning, deep learning, neural network, graph neural network, transformer, large language model, generative model, diffusion model, and reinforcement learning. Category 2 (Drug Discovery Applications): drug discovery, drug development, de novo drug design, virtual screening, drug repurposing, drug–target interaction, protein structure prediction, ADMET, and toxicity prediction. Category 3 (Clinical Translation): clinical trial, clinical translation, precision medicine, real-world data, and regulatory.

### 2.2. Inclusion and Exclusion Criteria

Studies were included if they met the following the criteria: (1) were original research or reviews (excluding editorials, preprints); (2) described an AI model applied to preclinical drug discovery; (3) reported quantitative performance metrics (e.g., AUC, R^2^) from either computational validation (benchmark datasets) or experimental validation (in vitro/in vivo). Papers with only qualitative descriptions or pure theory without quantitative drug-relevant validation were excluded.

### 2.3. Selection and Organization

Two authors independently screened titles/abstracts. Disagreements were resolved by discussion. Selected papers were grouped by pipeline stages (Section 3.1, Section 3.2, Section 3.3, Section 3.4, Section 3.5, Section 3.6, Section 3.7 and Section 3.8).

### 2.4. Model Selection for Tables

Representative models were chosen based on the following: (1) methodological innovation; (2) reproducibility/influence (open source or highly cited); (3) validation strength (external/experimental validation preferred; internally validated models included but noted in tables).

## 3. Applications of Artificial Intelligence in Drug Discovery

Based on the retrieved literature, the following sections synthesize the key applications of AI across the drug discovery pipeline. The drug development process begins with the identification of biological targets implicated in a disease, followed by the screening of potential lead compounds that may interact with the targets, lead optimization, and then proceeds to preclinical and clinical trials [3]. This chapter reviews the applications of artificial intelligence in the early stages of drug discovery, including drug–target interactions, protein structure prediction, de novo drug design, AI-assisted virtual screening, and evaluation of physicochemical properties and toxicity.

### 3.1. AI in Facilitating Drug–Target Interaction Prediction

The term drug–target interaction (DTI) refers to drug binding to a target, causing changes to target behavior or function with therapeutic effect [3,16]. Drug–target affinity (DTA) measures the interaction strength between a drug and its target and is a key criterion for drug candidacy [17]. DTI prediction methods are broadly categorized by their input data. DTI classification predicts whether a drug interacts with a target (binary classification), whereas DTA regression predicts the binding affinity between them [18]. Sequence-based methods use drug SMILES strings and target protein amino acid sequences as input, without requiring 3D structures [19]. While structure-based methods incorporate 3D structural information and capture spatial relationships [20]. Traditional methods like structure-based docking or ligand-based methods suffer from a high computational load, a lack of available three-dimensional structures, and an overreliance on known active ligands [21,22,23]. These limitations underscore the urgency of developing new exploratory methods to predict DTIs.

The DTI prediction problem can also be approached correctly with the aid of machine learning through two of its distinct yet complementary paradigms [24].

#### 3.1.1. Traditional Machine Learning Methods in DTI Prediction

Traditional machine learning methods claim that similarly acting drugs target similar receptors [24]. In an imbalanced dataset containing a target family, DTI prediction can be improved with random projection and NearMiss combined with a random forest method, where an AUC of 92.3 to 99.3% can be achieved across 4 dataset benchmarks [25]. A method called SS-HPBCT (semi-supervised impurity) by Alves et al. achieved 0.458 AUPRC and 0.425 MCC on the GPCR, which is over a 60% improvement over PBCT and was designed specifically to predict DTIs in partially labeled and imbalanced datasets [26]. Zhang et al. proposed an unsupervised noise-removal model called OpBGM (OPTICS + BGMM) that is able to locate potential drug-binding sites without requiring labeled data [27].

#### 3.1.2. Deep Learning Methods in DTI Prediction

Deep learning has further accelerated the progress of DTI by achieving end-to-end learning from the original molecular graphs and sequences. Topological and sequential patterns are captured by GNNs [28]/CNNs [29], and the recent diffusion-based generative model [30] captures docking as pose generation with more accuracy and speed. Recent developments show the prominence of several important trends.

Zeng et al. developed Drug-Online, a unified platform that integrates deep learning tools for drug–target interaction, affinity, and binding site prediction to streamline drug screening. However, its reliance on GNN-based models limits architectural diversity, and performance may vary across different datasets. However, like most DTI tools, Drug-Online remains at the in silico stage and awaits experimental validation [17]. Zhang et al. proposed DrugMAN (GAT + Mutual Attention Network), achieving superior cold-start generalization (the smallest AUROC drop), which can contribute to in silico drug discovery and repurposing, though prospective experimental validation is still needed to confirm its utility for novel targets [31]. He et al. proposed FDTIIT, the first model to address interaction information overlap via dependency trade-off, maximizing integrated interaction utilization [32]. Deep learning also complements ML for DTI/drug–target affinity (DTA) prediction. Yao et al. proposed SHGCL-DTI, which uses heterogeneous graph contrastive learning to augment supervised DTI prediction and alleviate data scarcity [33].

#### 3.1.3. Limitations and Prospection

Despite progress, deep learning-based DTI prediction faces hurdles. First, overconfidence in predictions remains problematic, as models often produce high-probability outputs even for unreliable predictions, leading to false positives in experimental validation [34]. Additionally, while AI excels at targeting well-characterized orthosteric pockets (e.g., the active sites of kinases), it exhibits poor predictive power for allosteric modulators and intrinsically disordered proteins (IDPs). These targets lack fixed three-dimensional structures before ligand binding but represent a massive, largely untapped frontier for treating complex diseases [35,36]. To address these hurdles, Bayesian deep learning enables uncertainty quantification, which helps filter unreliable outputs and reduce experimental false positives [37].

**Figure 2:**
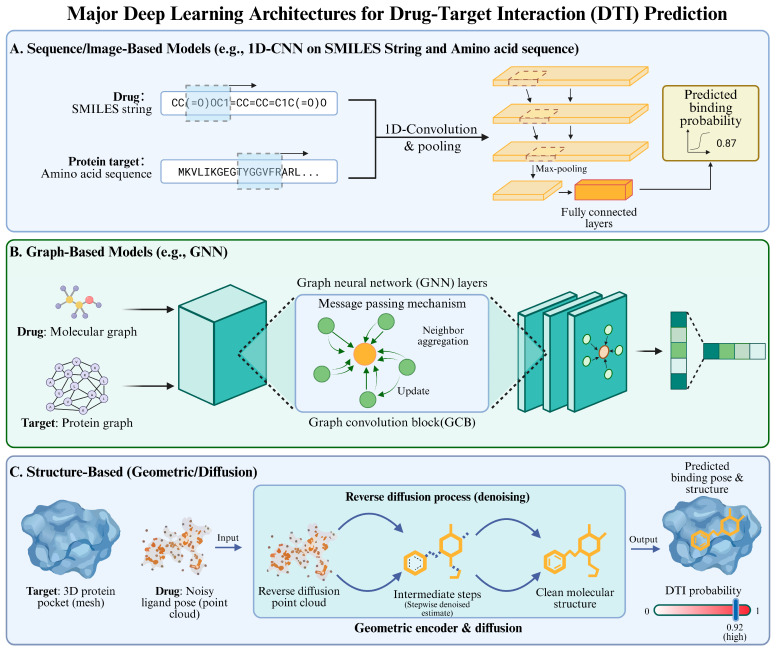
Major Deep Learning Architectures for drug–target Interaction (DTI) Prediction. (**A**) Sequence/Image-based Models. Drug SMILES and protein sequences are processed by 1D-CNNs. Convolutional kernels slide across inputs to extract local features, which are then pooled and passed through fully connected layers to predict binding probability. (**B**) Graph-based Models. Molecules are represented as graphs (nodes/edges). GNNs use message passing, where information from neighboring nodes is collected to revise the state of a node. This process captures the topological features necessary for affine predictions. (**C**) Structure-based Generative Models. Using Geometric Deep Learning, the models implement a reverse diffusion (denoising) process. Based on the protein’s 3D surface mesh, the model incrementally refines a noisy ligand point cloud into a legitimate, high-affinity, docked pose. Created in BioRender. Cai, M. (2026) https://BioRender.com/evbrk14 (accessed on 20 April 2026).

Figure 2 depicts key DL architectures for molecular representations, while Table 1 compares representative DTI models with their architectures, advantages, and applications.

**Table 1:** Summary of representative deep learning models for drug–target interaction (DTI) prediction.

Model	Underlying Model/Architecture	Authors & Publication Year	Main Applications (Guidance for Clinical/Drug Discovery)	Advantages/Innovations	Limitations	Validation Type	Dataset(s)	Experimental Validation	Translational Stage
DTIAM	Transformer + multi-task self-supervised (MLM, MDP, MFGP) + AutoML	Lu et al., 2025 [18]	DTI, DTA, MoA (activation/inhibition)	Unified DTI/DTA/MoA Self-supervised pre-training SOTA in cold start Identified TMEM16A inhibitor	Ignores protein dynamics, mutations, cellular environment	10-fold CV (DTI); 5-fold CV (DTA/MoA); warm/drug-cold/target-cold; external validation	DTI: Yamanishi_08, Hetionet; DTA: Davis, KIBA; MoA: TTD; Pre-train: GuacaMol (ChEMBL)	Yes (dehydrocostus lactone inhibits TMEM16A, IC50 = 111.97 nM, patch-clamp)	Preclinical (in silico + in vitro)
DrugBAN	Bilinear Attention Network (BAN) for local interactions Conditional Domain Adversarial Network (CDAN) Input: drug graphs + protein sequences	Bai et al., 2023 [38] Reusability: Tao Xu et al., 2024 [39]	Drug—target interaction (DTI) prediction Cross-domain prediction for novel drugs/targets (cold-start) Extended to cell line–drug response & mutation–drug association	BAN explicitly models local residue–atom interactions CDAN improves generalization to unseen domains Extensible to other bipartite networks	Performance depends on clustering method for domain split Cross-domain performance drops vs. in-domain	In-domain: random split Cross-domain: clustering split (drugs & proteins)	BindingDB, BioSNAP, Human	Original: None Supplement: Borneol study (2024) validated TRPA1/TRPM8 via patch-clamp, knockout mice, and clinical trial [40]	Original: preclinical (in silico) Supplement: advanced to clinical trial [40]
MolTrans (Molecular Interaction Transformer)	Transformer encoder FCS substructure mining Pairwise interaction map + CNN	Original: Kexin Huang et al., 2020 [41] Extension: Jesus de la Fuente et al., 2025 [42]	DTI prediction Virtual screening Drug repurposing	Mines interpretable substructures from massive unlabeled data Explicit pairwise interaction map for interpretability Robust performance with scarce labeled data	Fails on small datasets AUC < 0.5 under strict splits Poor to unseen protein families	Original: Internal validation (random split) Supplement: RMSD-based structure split (novel validation method)	BIOSNAP DAVIS BindingDB	Original: None Supplement: Cell viability assay, Western blot, Surface plasmon resonance [42]	Original: Preclinical (in silico) Extension: Preclinical (in vitro validated) [42]
TransformerCPI	Modified Transformer (encoder–decoder) GCN for compounds Gated CNN for proteins Self-attention	Lifan Chen et al., 2020 [43]	Virtual screening Lead optimization Targets without 3D structures (GPCRs, kinases)	Interpretable via attention maps Label reversal to reduce ligand bias Context-aware ligand features	Attention regions may not align with exact binding sites	Random split (Human, C. elegans) Cold-start split (BindingDB) Label reversal (GPCR, Kinase)	Human C. elegans BindingDB GPCR Kinase	Original: None Supplement: Cell lines, Patient-derived organoids, Animal models [44]	Original: Preclinical (in silico) Extension: Preclinical (in vivo) [44]
Chemical Space Neural Networks (CSNN)	Labels as Features (LaF) on chemical similarity graphs Training-free GNN/transductive graph classification	Hansson, F.G. et al., 2025 [28]	Predict hGPCR-drug interactions Discover off-target effects Prioritize DTIs for experiments	LaF enables training-free prediction (F1 up to 93%) Prevents OOD predictions (no neighborhood = no prediction) Single forward pass for all 128 GPCRs	Covers only 128 GPCRs Public data bias may overestimate homophily	Internal (train/val/test split); transductive	ChEMBL, IUPHAR/BPS, pdCSM-GPCR, custom yeast screen	Yes (partial): 14 novel DTIs validated in yeast	Preclinical (in silico + yeast)
BridgeDPI	Combines CNN, FFN, and GNN. Introduces “bridge nodes” to construct a supervised drug-protein association network (bridge graph).	Yifan Wu et al., 2022 [45]	Fast and accurate DPI prediction to assist lab experiments. Predicting interactions involving unseen proteins. Virtual drug screening and target identification.	Uses bridge nodes to construct a learnable drug-protein association network, which improves prediction for unseen proteins. Combines network-level information (guilt-by-association) with learning-based methods. Achieves SOTA results on multiple benchmarks.	Number of bridge nodes requires careful tuning (too many may cause overfitting and high computational cost). Lack of experimental validation.	Internal: Random split (training/validation/test). External: Trained on BindingDB, tested on DUD-E (independent set).	BindingDB (for main comparison) C. elegans HUMAN DUD-E (for independent test)	None (computational only)	Preclinical (in silico drug discovery)
KGE_NFM	Knowledge Graph Embedding (KGE) using DistMult Neural Factorization Machine (NFM)	Qing Ye et al., 2021 [46]	DTI prediction Protein cold-start scenarios	No similarity networks needed Best for new protein targets (e.g., 19% AUPR gain) Integrates multi-omics & fingerprints	Sensitive to hyperparameters KG quality matters (noisy nodes hurt) No experimental validation	10-fold cross-validation (warm start, drug cold-start, protein cold-start)	Yamanishi_08 BioKG	None (computational only)	Preclinical (in silico)

### 3.2. AI in Protein Structure Prediction

The primary molecular targets of therapeutic agents are proteins [21]. Therefore, accurate prediction of their three-dimensional structures is fundamental to understanding drug–target interactions and advancing drug design. Understanding their structures in three dimensions exposes structure-function relationships and helps to optimally tune medicinal chemistry to give molecular-level insights into the mechanisms of a disease [47,48]. X-ray crystallography, NMR, and cryo-EM are traditional experimental methods that are resource-intensive and subject to technical limitations [49]. AI has rapidly and dramatically changed the predictive landscape in structure determination.

DeepMind’s AlphaFold2 (AF2) utilizes a transformer-based [49] design, which applies mixed MSAs and contact maps in a multi-tier system to structure the evolutionary, physical, and geometric conditions of proteins. In the 14th iteration of the Critical Assessment of Protein Structure Prediction (CASP) competition, AF2 reached experimental-level accuracy among the participating predictors that incorporate an attention mechanism as a primary constituent of their learning architecture [50,51]. It has also been applied in the first stages of the drug discovery framework, as in the case of predicting the structure of the CDK20 protein in hepatocellular carcinoma, which resulted in 7 (seven) bioactive molecules post-docking [52]. Its main disadvantage is that it specializes in single-chain proteins and, therefore, cannot address multi-component bio-molecular complexes [53].

AlphaFold3 (AF3, 2024) increases its range to include all important biomolecules (proteins, DNA, RNA, ligands, and modified residues). It substitutes the Evoformer for a more straightforward Pairformer and incorporates a diffusion module to directly determine atomic coordinates from a noisy signal, allowing for conformational sampling [53,54]. AF3 is 1.5 times more accurate than traditional physics-based methods on the PoseBusters benchmark and exceeds most specialized methods for RNA structure prediction [54,55]. However, it still predicts static conformations similar to PDB and does not capture the dynamics of the solution [53].

ESMFold does not need to go through the multiple sequence alignments (MSAs), templates, and evolutionary databases, allowing for much higher speeds and resolutions [56,57]. Though ESMFold is somewhat less accurate than AF2 for well-studied proteins, it is better for orphan proteins and data-sparse targets, making it a useful tool for high-throughput peptide design [56,58].

RoseTTAFold employs a three-track neural network that concurrently processes sequence (1D), distance (2D), and coordinate (3D) information [59]. While AlphaFold often generates more diverse models, the top-ranked predictions of both methods show comparable fidelity [60].

Despite the remarkable progress in AI-based protein structure prediction, several critical limitations hinder its direct application in drug discovery. First, current AI tools predict static structures and miss dynamic changes from PTMs like phosphorylation [61]. Second, generalization to unseen ligands and complexes remains poor. Benchmarks show that docking accuracy drops sharply when ligands share low similarity (<0.50 Tanimoto) with training data, and allosteric sites are often misidentified as orthosteric pockets, indicating reliance on memorized patterns rather than physical principles [62]. Third, prediction of multi-chain complexes, particularly antibody–antigen interactions, remains unreliable, with failure rates exceeding 50% in low-homology scenarios [62,63]. These limitations underscore that AI-predicted structures must be critically evaluated through experimental or orthogonal methods before being used in therapeutic design.

Table 2 lists key protein structure prediction models with their architectures, applications, and limitations.

### 3.3. AI in De Novo Drug Design

With the ability to predict protein structures with high accuracy, AI now enables the generation of novel molecules tailored to these targets, a process known as de novo drug design. De novo drug design aims to generate novel chemical compounds based on target or ligand information [70]. By accelerating design cycles and improving decision-making, Machine learning, particularly deep learning, has significantly advanced modern de novo drug design [71].

#### 3.3.1. Classical Architectures in De Novo Drug Design

Generative models for this field can be broadly categorized into four classical architectures: recurrent neural networks (RNNs), generative adversarial networks (GANs), variational autoencoders (VAEs), and reinforcement learning (RL) [72,73]. RNNs generate molecules by probabilistically modeling sequences [72]. GANs employ a generator-discriminator game to create realistic structures [72]. VAEs learn latent representations for molecular generation [72,73,74]. and RL frames generation as an optimization problem guided by reward functions [75]. While each approach has demonstrated utility, such as target-directed design with conditional RNNs [76], scaffold generation with GANs [77], or multi-objective optimization with improved VAEs [78]. Despite their successes, these classical methods often suffer from inherent limitations: RNNs struggle with vanishing gradients and capturing long-range dependencies; GANs face training instability and mode collapse risks; VAEs tend to generate blurred samples with limited diversity; and RL approaches are sensitive to reward function design, hyperparameters, and suffer from reproducibility challenges [8,75].

#### 3.3.2. Emerging Models in De Novo Drug Design

Recent advances in generative AI have introduced powerful new architectures that overcome many limitations of earlier approaches. Diffusion models, originally developed for image synthesis, have been adapted for 3D molecular generation conditioned on protein pockets. Models such as DiffSBDD [79], PMDM [80], and DiffLinker [81] employ equivariant graph neural networks to progressively denoise atomic coordinates, producing physically realistic ligand poses with high binding affinity (see Table 3). These methods excel at generating molecules directly in three dimensions, preserving stereochemistry through equivariant architectures and enabling structure-based drug design across diverse scenarios.

Simultaneously, Large Language Models (LLMs) pretrained on massive chemical corpora have emerged as powerful generators. MolGPT uses a transformer–decoder architecture to generate SMILES strings with high validity and uniqueness, while supporting conditional generation on desired properties or scaffolds [82]. However, as Doron et al. emphasize, generation capability alone is insufficient; generative chemistry is most useful as part of an integrated platform [83] that combines AI generation with expert human judgment and experimental feedback to rigorously filter candidates.

A promising direction is persona-driven generative AI, which enhances AI–human collaboration by enabling AI systems to adopt specialized roles (e.g., regulatory specialist, clinical researcher) and interact in contextually appropriate ways across drug development, using contextual priming to improve domain-specific reasoning and communication in pharmaceutical applications [84].

In 2024, Insilico Medicine reported Phase IIa trial results for a TNIK inhibitor—the first AI-generated molecule to demonstrate clinical efficacy, with discovery-to-candidate timelines of just 18 months [85]. Beyond molecular generation, integrating generative AI with digital twins enables holistic patient simulations that capture both physiological and behavioral dimensions. As Wan et al. (2025) describe, persona-enhanced systems can generate synthetic patient profiles with realistic adherence patterns and quality-of-life preferences, complementing digital twin physiological modeling—allowing trial designers to explore both biomarker trajectories and patient-reported outcomes [84]. These agentic frameworks not only generate novel structures but also simulate decision-making processes.

Despite these successes, several key challenges persist, including ensuring managing computational costs and experimentally validating generated molecules. As highlighted by Zhang et al., incorporating physical principles into data-driven generative models is crucial to reduce data dependency and improve generalizability [86]. A related issue is reward hacking in reinforcement learning-based generation, where models exploit scoring-function loopholes to produce structures with high predicted scores but poor synthetic feasibility or limited biological relevance. This phenomenon arises when prediction models fail to extrapolate accurately for molecules that deviate substantially from the training data, leading to optimized yet impractical candidates [87]. Therefore, synthetic accessibility remains a key translational challenge, as many generated molecules lack practical synthetic routes or cannot be assembled from available building blocks [88]. Recent approaches have sought to address this by reframing synthesizability from a mere constraint into an active design objective, for instance, by incorporating a dedicated Synthesizability Estimation Model (SEM) that provides action masks to guide the generative process, operating alongside a multi-component reward function rather than embedding synthetic feasibility directly into the reward itself [89]. In addition, this issue can also be addressed by designing generative models that directly predict reaction products from reactants under reaction-type constraints, thereby ensuring that the generated molecules are grounded in real-world synthetic pathways [90]. Looking ahead, a paradigm shift toward “closing the loop” in drug discovery is envisioned, where generative AI integrates with automated high-throughput experimentation to create continuous cycles of hypothesis generation and validation. This vision extends to “lights-out” laboratories and the convergence of AI with synthetic biology, organ-on-chip technologies, and digital twins, promising to reshape R&D by enabling faster iteration and more rapid delivery of novel therapeutics [83].

**Table 3:** Overview of state-of-the-art generative models for de novo drug design.

Model	Underlying Model/Architecture	Authors & Publication Year	Main Applications (Guidance for Clinical/Drug Discovery)	Advantages/Innovations	Limitations	Validation Type	Dataset(s)	Experimental Validation	Translational Stage
DiffLinker	Based on Diffusion Models and E(3)-Equivariant Graph Neural Networks (EGNN)	Ilia Igashov et al., 2024 [81]	Used for connecting multiple molecular fragments in Fragment-Based Drug Design (FBDD); scaffold hopping; pocket-conditioned linker design	E(3)-equivariant; multi-fragment; auto linker length; pocket conditioning; fast sampling	OpenBabel post-processing; poor for long linkers (PROTACs);	Internal random split	ZINC, CASF, GEOM, Pockets	None (computational)	Preclinical (in silico)
PMDM (Pocket-based Molecular Diffusion Model)	Based on Diffusion Models, E(n)-Equivariant Graph Neural Networks (EGNN), and integrates SchNet with cross-attention mechanisms.	Lei Huang et al., 2024 [80]	Applied in Structure-Based Drug Design (SBDD) De novo 3D ligand generation Scaffold hopping Linker generation Lead optimization (Mpro, CDK2)	One-shot generation E(3)-equivariant Dual edges (covalent + vdW) Supports fragment inpainting without retraining 20× faster than autoregressive	Lower diversity than AR-SBDD/DiffSBDD	Internal (30% seq identity)	CrossDocked2020 (100 k train/100 test)	Main: None CDK2: Yes (in vitro enzyme assays)	Preclinical (in silico + early in vitro)
DiffSBDD	SE(3)-equivariant DDPM (Equivariant Denoising Diffusion Probabilistic Model)	Arne Schneuing et al., 2024 [79]	SBDD De novo generation Molecular inpainting Iterative optimization Negative design	SE(3)-equivariant (chirality-aware) Generates all atoms at once No retraining for new tasks Supports iterative improvement	De novo design challenging May generate disconnected molecules Over-represents small/macro rings No wet-lab validation	Original: Internal (sequence/EC-based splits) Supplement: External benchmarking (blind set, EC-based splits) [91]	CrossDocked, Binding MOAD	None (computational only)	Preclinical (in silico)
REINVENT 2.0	SMILES RNN + RL + diversity filter	Thomas Blaschke et al., 2020 [92]	De novo drug design; multi-parameter (ADMET, activity)	Balances exploration/exploitation Flexible scoring (sum/product) Transfer learning supported Op en-source production tool	Slow with predictive models	Original: Not systematically reported Supplement: retrospective validation used in some later studies [93]	ChEMBL + user custom	Original: None (computational only) Supplement: A later study (Thomas et al., 2024) included in vitro validation [94]	Original: Preclinical (in silico) Supplement: Preclinical (in vitro) [94]
MolGPT	Transformer–decoder (GPT) with masked self-attention	Viraj Bagal et al., 2021 [82]	De novo generation Conditional generation (properties/scaffolds) Lead optimization	First GPT for molecules High validity, uniqueness, novelty Precise multi-property & scaffold control Saliency map interpretability	Lower novelty on MOSES QED control overlap under scaffold constraints	Train/test split from MOSES and GuacaMol (test set specified)	MOSES, GuacaMol	None (computational only)	Preclinical (in silico)
Lib-INVENT	RNN encoder–decoder + RL (DAP)	Vendy Fialková et al., 2021 [95]	Scaffold decoration, focused library generation	Reaction-based slicing improves synthetic feasibility Reaction filters per attachment point Single prior reused via RL	Depends on user-provided reactions Small/invalid output if reactions inapplicable	Original: Internal: held-out Murcko scaffolds Supplement: Prospective case validation (LDHA inhibitor) [96]	ChEMBL27 (filtered, reaction-sliced)	Original: None (computational only) Supplement: In vivo active novel scaffolds [96]	Original: Preclinical (in silico) Supplement: Preclinical (lead optimization with in vivo validation) [96]
CReM	Matched Molecular Pairs (MMPs) Interchangeable fragment library Modes: Mutate, Grow, Link	Pavel Polishchuk, 2020 [97]	Lead optimization Exploring chemical space around a lead Scaffold decoration & fragment linking	Chemically valid by design Controls synthetic feasibility (via input SCScore) Adjustable context radius (1–5) Customizable fragment source	Cannot create new ring systems	Internal (benchmarking)	ChEMBL v22, DrugBank, Guacamol	None (computational only)	Preclinical (in silico)
RELATION	Encoder–decoder (3D CNN + LSTM), DSN for BiTL, CVAE, BO sampling	Mingyang Wang et al., 2022 [98]	Structure-based de novo design for AKT1, CDK2	3D complex input Bidirectional TL Pharmacophore + BO improves docking	Low validity (~30%) BO reduces novelty Long training (8 days)	Internal (validity, uniqueness, novelty, internal diversity, FCD, T-SNE, docking scores, pharmacophore scores)	Source: ZINC (1.2 M) Target: AKT1 (407), CDK2 (1017) inhibitors PDB: 4GV1, 4KD1	None (computational only)	Preclinical (in silico)
CogMol	VAE for SMILES CLaSS rejection sampling Protein sequence embeddings (UniRep-based)	Chenthamarakshan et al., 2023 [99]	De novo inhibitor design for protein targets (e.g., SARS-CoV-2 spike RBD, M^pro^)	Sequence-guided (no 3D structure/binders needed) 50% hit rate (2/4 per target) Broad-spectrum against VOCs	Binding predictor biased to µM inhibitors Low confidence for dissimilar targets Only 4 compounds per target tested (proof-of-concept)	Internal (training/test split) + experimental validation	VAE training: MOSES (~1.6 M molecules) + BindingDB (~211 K ligands) Binding predictor: BindingDB	Yes—in vitro enzyme assay (M^pro^), pseudovirus/live virus (spike), X-ray (M^pro^)	Preclinical (in vitro/viral models)

### 3.4. AI in Protein Design and Engineering

While the previous Section 3.3 focused on AI-generated small molecules that bind to protein targets, this section shifts the focus to the proteins themselves: using AI to design novel sequences that fold into desired structures and perform specific functions. Deep learning has revolutionized de novo protein design, enabling generative protein engineering.

#### 3.4.1. Inverse Folding: From Backbone to Sequence

Inverse folding in protein design predicts an amino acid sequence for a given 3D backbone structure. Traditional physics-based methods (e.g., Rosetta) are computationally expensive and often require extensive sampling [100]. Deep learning has transformed this landscape.

Dauparas et al. introduced ProteinMPNN, a message-passing neural network that predicts protein sequences from backbone coordinates using order-agnostic decoding and symmetry-aware features. It outperforms Rosetta in native sequence recovery (52.4% vs. 32.9%), experimentally rescues previously failed designs, and is over 200 times faster, though it lacks the physical transparency of energy-based models. This highly validated method provides a broadly applicable tool for designing monomers, cyclic oligomers, nanoparticles, and target-binding proteins [101]. LigandMPNN generalizes the ProteinMPNN architecture to incorporate nonprotein atoms, explicitly modeling all nonprotein components and their interactions with protein side chains. This deep learning model designs protein sequences around small molecules, nucleotides, and metals—a critical capability for designing enzymes, sensors, and binding proteins. It has been experimentally validated to rescue failed designs and improve binding affinity by up to 100-fold. However, caution is advised for ligands with rare chemical elements, and hybridization with physically based approaches may be beneficial in low-data regimes [102].

#### 3.4.2. Generative Models for Protein Backbone Design

The next frontier is generating entirely new protein backbones that do not exist in nature. This is where generative AI, particularly diffusion models, has made remarkable strides.

RFdiffusion, built upon the RoseTTAFold architecture, inverts the structure prediction process. Starting from random noise, it iteratively denoises to produce diverse, physically plausible protein structures. The model can be conditioned on various constraints, such as symmetric assemblies, functional motifs, or binding targets. Its power was dramatically demonstrated in the design of novel protein binders against difficult targets, including the successful engineering of proteins that neutralize elapid snake venom toxins with nanomolar affinities and crystal structures nearly identical to the computational models [103,104]. However, a current limitation is that RFdiffusion is unable to explicitly model bound small molecules [103].

#### 3.4.3. Sequence-Structure Co-Design and Multi-Objective Optimization

Recognizing that sequence and structure are interdependent, advanced models now perform co-design, simultaneously generating both the backbone and the sequence.

ESM3 is a multimodal generative language model that unifies protein sequence, structure, and function for prompt-based design. It experimentally validated its capability by generating a novel fluorescent protein, esmGFP, which shares only 58% sequence identity with known fluorescent proteins, equivalent to simulating over 500 million years of evolution. Despite this success, ESM3 has limitations: complex tasks often require fine-tuning to improve fidelity, and not all prompts guarantee functional proteins without experimental validation. Interpretability is provided through learned representations that capture biological structures and functions from evolutionary data. These features underscore AI’s role in exploring vast sequence spaces and proposing novel proteins [105].

For multi-objective optimization, frameworks like SAGE-Prot combine generative models with predictive property models in an iterative loop. Applied to TEM-1 β-lactamase, it generated variants with up to 752-fold increased enzymatic activity while maintaining stability, illustrating how AI-driven generative exploration can accelerate functional protein engineering. The modular architecture separates generation, diversification, and evaluation, enhancing interpretability and control. However, performance declines for proteins longer than 300 residues, and QSPR prediction accuracy remains modest for certain properties, limiting optimization resolution [106].

#### 3.4.4. Limitations and the Future of Protein Engineering

Despite recent advances, key challenges still limit the scope and reliability of current AI-based protein design methods. This activity gap stems from limitations including the lack of suitable protein scaffolds, the complexity of native protein sequence-structure relationships, and the difficulty of achieving high catalytic activity directly from computational design [107]; moreover, even state-of-the-art methods like RFdiffusion2, despite achieving comprehensive in silico success on benchmark active sites, yield experimentally validated enzymes with catalytic efficiencies below those of native enzymes, suggesting that current theozyme-based conditioning may not capture all interactions necessary for high activity [108]. Recent advances are beginning to address these limitations through complementary strategies. For instance, explicit modeling of full reaction coordinates has proven effective, with Lauko et al. demonstrating that screening designs for active site preorganization across each step of multistep catalytic cycles dramatically improved success rates, yielding serine hydrolases with catalytic efficiencies up to 2.2 × 10^5^ M^−1^s^−1^ [109]. This shift from static to dynamic design, which stabilizes entire reaction trajectories, suggests that mastering conformational landscapes is key to future breakthroughs.

In parallel, integrating protein language models with automated biofoundries is addressing the bottleneck of experimental throughput, enabling closed-loop systems that execute the Design–Build–Test–Learn cycle autonomously. Platforms like PLMeAE combine ESM-based zero-shot mutation prediction with automated biofoundry operations to accelerate protein engineering. Applied to engineer an aminoacyl-tRNA synthetase, PLMeAE completed four rounds of evolution within 10 days, yielding mutants with up to 2.4-fold improved activity [110].

### 3.5. Artificial Intelligence in Virtual Screening

**Figure 3:**
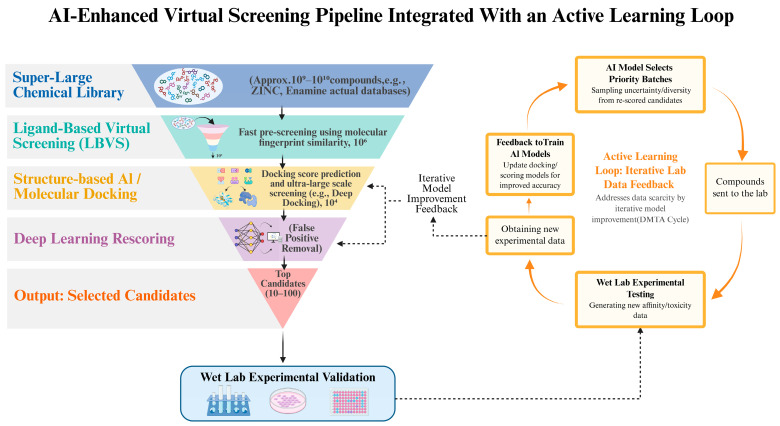
AI–Enhanced Virtual Screening Pipeline Integrated With an Active Learning Loop. (**Left**) The hierarchical screening workflow for ultra-large chemical libraries, comprising sequential stages of Ligand–Based Virtual Screening (LBVS), Structure–based AI (Deep Docking), and Deep Learning Rescoring. (**Right**) The Active Learning (AL) cycle showing the integration of experimental feedback into the iterative Design–Make–Test–Analyze (DMTA) process. Dashed lines indicate the flow of wet–lab data for model retraining and refinement. Created in BioRender. Cai, M. (2026) https://BioRender.com/yspr55x (accessed on 20 April 2026).

Once designed, these proteins and their potential ligands must be efficiently screened, leading us to AI-enhanced virtual screening (VS). VS is a core strategy for identifying potential drug candidates from large chemical libraries [3,111]. While traditional VS faces bottlenecks in efficiency and accuracy when handling ultra-large libraries containing billions of compounds [112,113]. AI integration fundamentally reshapes this landscape through a hierarchical workflow (Figure 3).

#### 3.5.1. AI in Ligand-Based Virtual Screening (LBVS)

In LBVS, static fingerprints are being superseded by AI-driven semantic representation learning. Traditional similarity searching is now superseded by Transformer-based language models and contrastive learning frameworks [114,115]. As highlighted in recent discovery campaigns, architectures like Directed Message Passing Neural Networks (D-MPNNs, as implemented in Chemprop) utilize advanced graph neural networks (GNNs) to embed molecular topology and geometry directly into continuous latent spaces [116]. By learning continuous, context-aware representations directly from molecular graphs, these models can capture subtle structural relationships that enable scaffold hopping—effectively identifying functionally similar but structurally novel compounds that would be missed by Tanimoto-based similarity searches [117,118].

#### 3.5.2. AI in Structure-Based Virtual Screening (SBVS)

For SBVS, AI transcends the speed–accuracy trade-off of physical docking. Current Deep Docking protocols utilize deep neural networks to approximate docking scores across massive libraries without exhaustive simulation [119]. More critically, AI-driven rescoring functions have revolutionized classification. Recent architectures, such as RTMScore, learn a statistical potential based on residue-atom distance likelihood, substantially outperforming conventional scoring functions in virtual screening benchmarks [120]. By accurately prioritizing true binders in virtual screening benchmarks, these models significantly mitigate the false-positive rates that have historically limited the success of large-scale computational campaigns.

#### 3.5.3. Active Learning and Commercial Implementation

Ultimately, the workflow culminates in an “Active Learning” cycle, realizing the concept of “Smart Data”. As depicted in the right panel of Figure 3, this is no longer a linear process but a “Lab-in-a-loop” system. AI models iteratively select small batches of top-scoring candidates for experimental testing; the resulting data is fed back to retrain the model, progressively refining its predictive power [121]. This strategy has been successfully implemented in virtual screening campaigns against diverse targets. For instance, GEM-Screen utilized an active learning strategy by training on docking scores of a small fraction of a compound library to approximate docking outcomes for yet-unprocessed entries, successfully enriching more than 90% of hit scaffolds from billion-scale libraries while docking only a small subset [122].

The maturity of these AI methodologies has led to the emergence of powerful commercial platforms. Companies such as Iktos have integrated generative AI with a chemistry-aware design approach that builds molecules step by step using known reactions and real starting materials, ensuring the synthetic accessibility of generated molecules, thereby enhancing the practical utility of virtual screening by prioritizing compounds that are both novel and readily synthesizable for experimental validation [123]. Atomwise’s AtomNet^®^ platform leverages deep convolutional neural networks to screen vast chemical spaces, successfully identifying novel hits across diverse protein classes and demonstrating that computational virtual screening can achieve sufficient accuracy to serve as a primary discovery tool [124].

#### 3.5.4. Challenges and Future Directions

Despite recent advances, deep learning-based binding pose prediction models have yet to outperform traditional physics-based methods in pocket-oriented docking tasks, as they often inadequately consider receptor pocket flexibility and struggle to predict precise receptor–ligand interactions with high pose accuracy [125]. Even after AI-based prioritization, a large proportion of nominated compounds fail in subsequent experimental validation [119]. Future efforts should focus on closing the virtual-experimental gap through iterative feedback loops, where AI predictions are continuously refined by experimental outcomes. Beyond these, AI-based virtual screening remains hindered by high false positive rates. Models with high AUC on retrospective benchmarks often fail to translate into experimental hit rates, as benchmark datasets are enriched with active compounds and do not reflect the extreme class imbalance of real screening libraries [126,127]. Current solutions to address the high false positive rate in AI-based virtual screening include: leveraging active learning strategies to iteratively improve the quality of training data [128]; adopting tailored loss functions such as Focal Loss to mitigate class imbalance at the algorithmic levels [129]; using vScreenML 2.0 as a post-docking classifier that reduces false positives and outperforms conventional scoring functions [127].

### 3.6. AI in Drug Repurposing

Beyond screening novel compounds, AI also excels at finding new uses for existing drugs, a strategy known as drug repurposing. Drug repurposing, the identification of new therapeutic indications for existing drugs, has emerged as a strategic alternative to traditional de novo drug development, offering reduced timelines, lower costs, and established safety profiles [130]. However, traditional drug repurposing lacked the tools to systematically interrogate large-scale multiomics data. AI now enables unbiased data integration and network-based analysis to uncover hidden drug–target–disease relationships [131].

AI-driven drug repurposing leverages several complementary paradigms. First, signature-based approaches compare disease-specific molecular signatures (e.g., gene expression profiles) with drug-induced signatures to identify compounds that reverse pathological states [132,133]. Second, target-centric methods predict off-target interactions for approved drugs, revealing novel mechanisms of action [134,135]. Third, genetic and knowledge graph-based techniques leverage large-scale biomedical ontologies and real-world data to infer drug-disease associations [136]. Table 4 summarizes representative AI models for drug repurposing. The utility of these AI-driven paradigms is illustrated by several recent studies. For example, CRISP demonstrates drug repurposing by zero-shot prediction of sorafenib’s efficacy in leukaemia from a solid-tumour-trained model, a finding validated by independent studies confirming CXCR4 inhibition as the key mechanism [137].

Despite these successes, AI-driven repurposing faces significant translational hurdles beyond computational accuracy. A notable example is the work by Gacem et al., who used the SPOKE knowledge graph to identify bavisant as a therapeutic candidate for multiple sclerosis. Although bavisant promoted remyelination and neuroprotection in multiple preclinical models (including LPC, cuprizone, and humanized chimeric mice), it failed to significantly improve clinical scores in the MOG-induced EAE model when administered during the chronic phase, despite its known safety profile [138]. This discrepancy highlights a key limitation in current AI-driven drug repurposing: predictions are often based on preclinical models that incompletely recapitulate human disease pathology, and may overlook critical factors such as therapeutic timing and disease heterogeneity. It underscores the need to integrate more predictive translational models, early-stage experimental validation, and consideration of regulatory and clinical endpoints into AI pipelines to mitigate late-stage failures.

Beyond the successes in identifying novel therapeutic uses for existing drugs, a critical but often overlooked issue is data leakage in transductive graph-based repurposing models, where node embeddings are generated before train–test splitting, artificially inflating performance [42]. This issue is further illustrated in matrix-oriented collaborative filtering, where training fails to distinguish unknown associations from those masked for testing, similarly leading to inflated performance [139]. The consequence is benchmark inflation: reported metrics such as AUC often overestimate a model’s true ability to generalize [139]. Even widely used splitting methods fail to ensure sufficient separation between training and test sets, causing models to succeed via memorization rather than genuine generalization [140]. Addressing these issues requires inductive evaluation strategies, such as time-split validation or cold-start splits, where test drugs or diseases have no prior evidence in the training set [42,139]. Evaluating model performance on out-of-distribution samples is also essential to avoid overestimation and to reflect real-world predictive capability [140].

Beyond evaluation methodology, successful repurposing must also contend with biological complexity. Many approved drugs work through multi-target mechanisms, and the integration of network pharmacology with artificial intelligence is increasingly recognized as essential for tackling the complexity of diseases like cancer and neurodegeneration [141,142]. However, a repurposed drug might hit the primary target but simultaneously trigger adverse off-target effects that were acceptable for the original indication but toxic in the new patient population. Therefore, robust experimental validation must go beyond simple binding assays. Future repurposing pipelines should prioritize phenotypic screening (e.g., using patient-derived organoids) to validate the holistic therapeutic response, ensuring that the “new use” does not introduce unforeseen safety risks.

**Table 4:** Summary of representative AI models for drug repurposing.

Model	Underlying Model/Architecture	Authors & Publication Year	Main Applications (Guidance for Clinical/Drug Discovery)	Advantages/Innovations	Limitations	Validation Type	Dataset(s)	Experimental Validation	Translational Stage
XG4Repo (eXplainable Graphs for Repurposing)	Path-based KG completion (AnyBURL + RNNLogic)	Jiménez, A. et al. (2024) [143]	Drug—disease prediction Explainable paths (genes, pathways) Candidate validation support	Interpretable (human-readable paths/rules) Learns metapaths automatically Adaptable to any biomedical KG Optimized for “treats” relation	Potential biases in model/dataset. Explanations can be improved. Only tested on Hetionet.	Random 80/10/10 split	Hetionet (2.25 M triples)	None (in silico only)	Preclinical (screening tool)
MNPTD Framework for CNS Drug Repurposing	Transcriptomics (limma) PPI (STRING) MNPTD gene scoring Drug-gene (DGIdb) RWR + proximity ML BBB (Random Forest, 95.7% acc, AUC 0.992) MedChem (CNS-likeness, tractability, safety)	Akguller, O. et al. (2025) [144]	Prioritize AD repurposing candidates Temporal dynamics-based recommendation Guide medchem optimization	Bridges computation & pharma development Evidence classification (established/clinical/mechanistic/speculative) Modality-specific ranking 80.1% approved drugs (0–1 yr)	No experimental validation Relies on literature curation and may not capture recent experimental data CNS filters may miss novel modalities BBB ML less reliable for peptides/biologics	Internal cross-validation (bootstrap, permutation) Train–test (80/20) for BBB ML Benchmark vs. known CNS drugs	GEO (GSE48350, GSE5281) STRING DGIdb (24,474→8247 CNS) 110 drugs for BBB training	None (computational only) Need AD model validation	Preclinical stage (all require AD model validation)
In-mac + ReBrain: A Network-Driven Drug Repurposing Framework for Brain Cancers	in-mac: SAR + Monte Carlo (1000 models/molecule) ReBrain: Network (Euclidean distance, regression) 15 brain-cancer-related pathway components	Prakash, O. (2026) [145]	Repurpose FDA drugs for brain cancers Compare multi-pathway profiles vs. reference drugs	Disease-specific, multi-pathway profiling Interactive network (knock-in/out, sliders) 70–95% ML accuracy Considers BBB Open-access web tool	Bound profile constraints Lack of sufficient experimental data	Internal: 5 ML models External: independent set (1392 molecules)	2809 FDA drugs (MW ≤ 500 Da) 15 brain-cancer components External: 1392 molecules (3 classes)	None (computational only)	Preclinical (in silico)
Automated Biological Evidence Generation Framework for Drug Repurposing	Pipeline: AnyBURL (rule learning) + multi-stage automatic filtering	Sudhahar, S. et al. (2024) [146]	Generate interpretable evidence chains for drug-disease predictions. Support preclinical decision-making.	Automatically filters uninformative paths (e.g., 95% reduction for Parkinson’s). Produces human-readable paths via genes/pathways. Validated against transcriptional changes in FXS mouse model.	Generates too many evidence chains for human review. Uses only public, proprietary, and curated data sources. No benchmark dataset for quantitative validation.	External experimental (in vivo)	Healx KG (proprietary + public: ChEMBL, DrugBank, KEGG, etc.); FXS mouse RNA-seq	Yes (in vivo FXS mouse model; Sulindac & Ibudilast)	Preclinical (in vivo)
TranSiGen	Variational autoencoder (VAE) based framework with self-supervised representation learning	Tong, X. et al., (2024) [132]	Phenotype-based drug discovery; ligand-based virtual screening; drug response prediction; drug repurposing (e.g., pancreatic cancer)	Denoises transcriptional profiles Outperforms baselines in DEG prediction for new compounds/cell lines Enables screening of novel scaffolds In vitro hit rates 38–80% (pancreatic cancer)	Performance drops when perturbation too subtle (R^2^ > 0.8) Early fusion suffers curse of dimensionality	Internal (chemical-blind, cell-blind splits)	LINCS L1000; CTRP; PRISM; TCGA	Yes (in vitro on YAPC cells; top-50 compounds)	Preclinical (in silico + in vitro)
UKEDR	KG embedding (PairRE) + pre-training (CReSS for drugs, DisBERT for diseases) + AFM recommender	Li, K. et al., (2025) [147]	Drug-disease prediction; cold-start; clinical trial simulation	Solves cold-start for new drugs/diseases; fuses KG and pre-trained features; largest KG (2.3 M entities)	KG construction labor-intensive and may introduce inconsistencies; integration of heterogeneous data remains challenging; high computational resources required	5-fold CV; cold-start scenarios	RepoAPP, RepoClin, RepoData; custom KG	None	Preclinical (in silico)

### 3.7. AI in Other Drug Properties

In addition to repurposing, AI plays a crucial role in predicting other key drug properties such as toxicity and physicochemical characteristics.

#### 3.7.1. AI in Prediction of Drug Toxicity

Toxicity describes how a certain chemical substance or a mixture can cause damaging effects to biological systems or to specific organs [148]. In the drug development pipeline, predicting the toxicity of a compound is a fundamental process. The most important aspect of toxicity prediction is determining the likelihood and type of adverse reactions a drug may cause. This guides researchers to molecular tailoring to remove or reduce the possibility of adverse effects [148]. Conventional toxicity predicting focuses on the use of cell and animal models. These models are expensive, time-consuming, ethically concerning, and in addition [149], they can yield contradictory results across different species [150]. Several studies have demonstrated that ML models trained on large datasets can achieve—or even surpass—the performance of conventional animal testing in predicting specific toxicities [149]. For instance, Jiang Lu et al. developed the ToxACoL model based on associative correlation learning, which models endpoint associations through graph topology and facilitates knowledge transfer via graph convolutional networks. ToxACoL significantly improves the prediction accuracy for data-scarce endpoints and reduces the required training data for sparse endpoints by 70–80%. It demonstrates greater robustness compared to representative baseline models, aids in the identification of structural alerts, and helps elucidate the underlying mechanisms of acute toxicity [151]. Deepak Rawat et al. developed an optimized ensemble model named OEKRF by combining eager random forest and sluggish K-star algorithms. Under three different scenarios, the optimized ensemble model performed favourably compared to other models. It achieved an 8% improvement in accuracy over the best-performing K-star machine learning model and a 21% improvement over the deep learning model AIPs-DeepEnC-GA. This model is capable of adapting to abrupt changes in dynamic environments and can learn from historical data to enhance generalization to unseen data [152].

#### 3.7.2. AI in Prediction of Physicochemical Properties

Physicochemical properties are inherent characteristics of drugs, and understanding them is essential for elucidating drug mechanisms of action and constructing predictive models. Solubility is regarded as a critical physicochemical parameter due to its influence on pharmacokinetic properties and formulation design [2]. Ahmadreza Roosta et al. integrated ML with atom contribution (AC) techniques to develop and evaluate two models, LSSVM-AC and MLPANN-AC. The LSSVM-AC model is based on the least squares support vector machine (LSSVM), constructed using a radial basis function kernel (RBF) and the coupled simulated annealing (CSA) algorithm. The MLPANN-AC model combines a multilayer perceptron artificial neural network (MLPANN) with the Levenberg–Marquardt algorithm. The use of the AC technique explains its novelty because it captures molecular details at the atomic level so that the models explain better the role of specific atoms that affect the solubility of supercritical CO_2_ (SC-CO_2_). The LSSVM-AC model solubility estimation was highly accurate with an R^2^ value of 0.99 and an AARD% of 7.20 [153]. The development of monoclonal antibodies (mAbs) and their commercialization, as well as their application in the treatment of cancer, infectious diseases, and autoimmune diseases, is done with consideration for the biophysical properties of the mAbs. I-En Wu et al. formulated a deep learning model called DeepSP and, for this purpose, developed 24 predictive models for 12 biophysical properties (6 based on Molecular Dynamics (MD) simulations and 6 based on DeepSP). The team developed an excellent predictive system in the biophysical characterization of significant parameters associated with mAb binding affinity, such as ELISA titer and BVP eigenvalues. While models based on DeepSP features performed a little worse than models based on MD features, the results were still satisfactory. DeepSP’s most noteworthy benefit is that it quickly extracts important features for model training for ADMET property prediction without being hindered by time constraints, lack of resources, or unstable results, which remain prevalent in more traditional approaches to MD [154].

#### 3.7.3. Limitations and Future Directions

Despite advances, AI models for ADMET prediction face bottlenecks. Models trained on conventional small molecules often fail on emerging modalities like PROTACs. Zhang et al. demonstrated that traditional ADMET models perform poorly on PROTACs, whereas models trained on PROTAC-specific data significantly outperformed them for key parameters. However, prediction accuracy for volume of distribution (Vss) remains modest, underscoring the need for modality-specific training data and tailored approaches [155]. To overcome these challenges, future work should focus on incorporating more sophisticated molecular features (e.g., structural biology data), leveraging transfer learning to broaden applicability beyond specific targets (e.g., EGFR-VHL system), and integrating larger, more diverse datasets to enhance model generalizability.

Second, difficulty in recognizing chirality within SMILES-based representations can affect ADMET prediction reliability for chiral compounds. Yoshikai et al. showed that Transformers struggle to distinguish enantiomers, frequently confusing ‘@’ and ‘@@’ markers, causing prolonged learning stagnation. This suggests ADMET models using such representations may fail to differentiate enantiomers with distinct ADMET profiles. Adopting Pre-Layer Normalization (pre-LN) architecture effectively mitigates chirality recognition difficulties in Transformers by accelerating learning and stabilizing training, enhancing reliability for chiral compounds [156].

Third, pretraining task design in graph transformers faces significant limitations. Kim et al. investigated pretraining strategies for molecular representation learning and revealed that previous pretraining tasks often fail to identify meaningful training targets. Their work demonstrated that integrating quantum-chemistry simulations as pretraining targets, rather than relying on simpler 2D descriptors, substantially improves downstream ADMET prediction performance [157].

Table 5 describes exemplifying AI models and pertinent features for ADMET (Absorption, Distribution, Metabolism, Excretion, and Toxicity) property prediction.

### 3.8. Integrating AI-Driven Drug Discovery with Clinical Practice

Despite significant progress in early-stage drug discovery enabled by AI, its integration into clinical practice remains limited: predictions are difficult to embed directly into clinical workflows [169,170], real-world data remains underutilized [170], the integration of patient-specific information is limited [171], and decision support tools are lacking [172]. This disconnect leaves many AI-predicted candidates at the computational stage, failing to reach patients.

**Figure 4:**
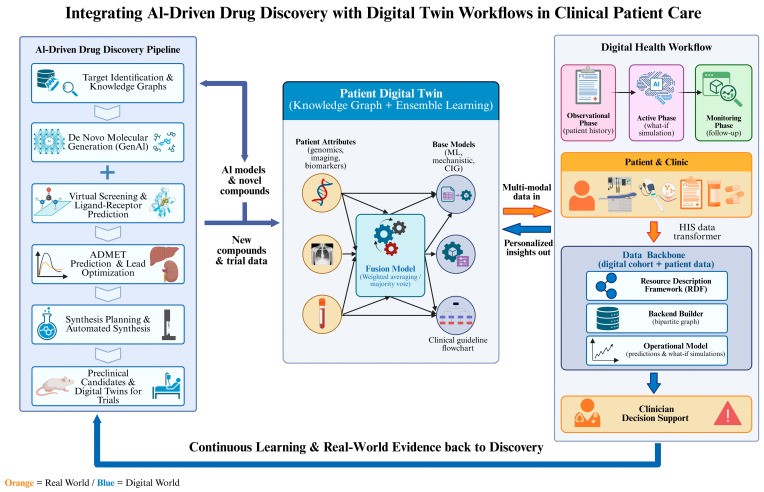
Integrating Al-Driven Drug Discovery with Digital Twin Workflows in Clinical Patient Care. The framework consists of three primary modules: the AI-driven drug discovery pipeline (**left**), which spans from target identification to preclinical digital twin trials; the Patient Digital Twin (**center**), which utilizes ensemble learning and fusion models to integrate multimodal patient attributes and clinical guidelines; and the clinical practice workflow (**right**), providing clinician decision support through a structured data backbone. Bidirectional arrows represent the exchange of AI models, novel compounds, and clinical trial data. The bottom feedback loop illustrates the continuous integration of real-world evidence back into the discovery phase. Colour coding: orange elements represent the real-world environment, and blue elements represent the digital domain. Created in BioRender. Cai, M. (2026) https://BioRender.com/xldgbt2 (accessed on 20 April 2026).

To bridge this gap, it is necessary to integrate AI more deeply into clinical applications at three levels (Figure 4). At the clinical workflow level, it is necessary to convert the AI prediction results into a form that is easy for clinicians to understand and use. ABIET, a Transformer-encoder model, uses attention weights to identify functional groups crucial for drug–target interactions, enabling medicinal chemists to understand which molecular substructures drive biological activity and accelerating clinical translation by bridging AI predictions with human decision-making [173]. Also, real-world data (RWD) must be integrated into the training and validation loops of AI models. RBD can not only compensate for sampling biases inherent in traditional databases but also capture long-term effectiveness, rare adverse events, and patient heterogeneity [174,175]. The BiGRU model, trained on real-world EHR data from 15 million patients, predicts progression from mild cognitive impairment to Alzheimer’s disease with high accuracy, enabling early risk stratification and accelerating clinical translation [176]. At the level of the clinical decision support system, AI predictions need to be translated into interactive clinical tools. For instance, the CONCERN Early Warning System, a machine learning model analyzing nursing documentation patterns, facilitates clinical translation by presenting its risk predictions as simple, non-intrusive colour-coded icons directly in the clinician’s existing workflow, which enhanced shared situational awareness and significantly reduced in-hospital mortality in a randomized trial [177]. Table 6 summarizes representative AI models that facilitate clinical translation through integration with clinical workflows.

## 4. Limitations and Solutions of AI in Clinical Translation

Despite progress in AI-driven early drug discovery, no AI-discovered drug has yet received regulatory approval [188]. Across the models surveyed in this review, the vast majority remain at the in silico stage, with only a few having undergone experimental validation. This translational chasm arises from seven interconnected limitations.

### 4.1. Data Quality and Bias: The Input Bottleneck

Building upon the baseline inflation and data leakage issues identified in previous sections, the foundational hurdle at the industry level remains the inherent bias of training data. Public databases suffer from data sparsity in rare diseases, which challenges the training of fit-for-use models [189], and implicit biases skew model training based on specific functional groups or non-sequence features [190]. Furthermore, databases predominantly contain successful experimental outcomes, while negative data remain largely unpublished [191]. This publication bias systematically drives models to overestimate real-world success rates. Proprietary data silos further prevent collective learning [192]. While cross-pharma initiatives like the MELLODDY project (utilizing federated learning across 2.6+ billion data points without compromising confidentiality) demonstrate technical feasibility [193], significant challenges such as “domain shift” [194] and the lack of robust legal frameworks remain [192].

### 4.2. Model Validation: The Dual Challenge of Generalization and Calibration

Moving from data to evaluation, over-optimistic benchmarking remains a pervasive issue. Models are frequently evaluated retrospectively on curated datasets that do not reflect the difficulty of real screening campaign [195]. Consequently, existing models often suffer from poor generalization when faced with out-of-distribution (OOD) data, such as novel scaffolds or unseen biological targets in practical scenarios [196]. This vulnerability arises because many AI models fail to learn the underlying physics of protein-ligand interactions, instead merely memorizing patterns from training data, which ultimately leads to unreliable predictions for truly novel targets [197]. Second, many machine learning models, particularly complex ensemble methods, suffer from poor calibration. A well-calibrated model ensures that predicted probabilities correspond to observed event rates: for instance, among patients assigned a 20% predicted risk, approximately 20% should experience the outcome [198]. A model might correctly rank patients (high C-index) but consistently overestimate or underestimate absolute risks [199]. This stems from model instability: risk estimates derived from small datasets can fluctuate based on the sample used, leading to miscalibration [200]. Therefore, while maintaining good discrimination performance, it is also essential to ensure good calibration and robust prospective generalization; future model development should focus on achieving this balance across diverse clinical scenarios.

### 4.3. Experimental Confirmation: The Validation Bottleneck

The transition from computational prediction to experimental validation introduces profound physical bottlenecks. Even if a model correctly ranks compounds mathematically, physical testing in vitro is fraught with biological assay artifacts. High-scoring virtual hits frequently fail in wet-lab environments due to prevalent interference mechanisms such as colloidal aggregation, chemical reactivity (including covalent modification and redox cycling), metal chelation, or by acting as Pan-Assay Interference Compounds (PAINs). These nuisance compounds generate artifactual readouts rather than reflecting genuine target engagement, thereby derailing the validation process [201,202].

To overcome these physical artifacts, recent studies have introduced computational filters that triage nuisance compounds before experimental testing. Abou Hajal et al. developed the BAD molecule filter to detect colloidal aggregators using machine learning [203]. Beyond rule-based filters, machine learning frameworks such as E-GuARD have been developed to predict assay interference through data augmentation and self-distillation, offering a complementary strategy to triage nuisance compounds before experimental testing [204].

### 4.4. Chemical Feasibility: Synthetic Accessibility and Medicinal Chemistry Realism

For generative models, the ‘reward hacking’ phenomenon detailed earlier frequently yields unstable or overly complex molecules [87]. Even when a synthetic route appears feasible in principle, its transition from milligram-scale laboratory synthesis to kilogram-scale manufacturing poses distinct challenges. Scalability demands not only a viable route but also considerations of yield optimization, purification efficiency, and cost of starting materials. A theoretically sound route may rely on hazardous reagents or purification steps that are impractical at industrial scale [205].

The physical form of a drug candidate strongly influences bioavailability, stability, and manufacturability. While computational models can now predict crystal structures, current methods are mostly limited to Z′ = 1 systems and DFT energy differences can vary by 0.5–1.0 kcal/mol, making reliable stability ranking difficult [206].

Thus, predicting a high-affinity binder conceptually does not guarantee it can be synthesized or scaled up. Despite its powerful predictive capabilities, AI is not a panacea; its outputs must be critically evaluated by experienced researchers for synthetic feasibility, cost-effectiveness, and practical implementation conditions. This paradigm shifts the scientist’s role from trial-and-error experimentation to enforcing medicinal chemistry realism and providing final human oversight.

### 4.5. Disease Relevance: Navigating Biological and In Vivo Complexity

Even if a molecule successfully passes basic experimental validation, a deeper translational gap remains: biological complexity. AI models are often trained on simplified representations, and their predictions frequently fail to translate into in vivo activity due to the complexity of biological systems [188]. AI models trained on such simplified in vitro data fail to capture the dynamic complexity of human physiology, widening the translational gap in drug development [207]. Emerging platforms are now transcending this limitation, moving beyond static in vitro assays toward tissue-level dynamics, inter-individual variability, and in vivo physiological contexts, as exemplified by PDGrapher (predicting combinatorial therapeutic targets via causal network modeling to reverse disease phenotypes) [208], GENEVA (capturing patient heterogeneity through pooled three-dimensional cultures and xenograft models) [209], and Squidiff (simulating organoid development and cellular responses to perturbations as a foundation model for the virtual cell) [210]. Continued integration of multi-scale data with advanced AI architectures will be essential to ultimately achieve precise prediction of in vivo drug responses.

### 4.6. Clinical Endpoints: Patient Heterogeneity and Translational Efficacy

A fundamental challenge in AI-powered drug discovery lies not in the algorithms themselves but in the heterogeneity of the patient populations that they must model. Inter-individual variability in genetics, environment, and comorbidities profoundly influences drug response [211,212]. In the context of a Phase II ketamine trial, these models yielded poor predictive performance, with AUC values falling to 0.27 and 0.29—effectively performing no better than random chance. This failure stems from the models’ tendency to overfit to noise in heterogeneous datasets, often producing predictions that are inversely correlated with the true outcomes [211]. This illustrates that, without effective patient stratification, even advanced machine learning models struggle to reliably identify true treatment effects from highly heterogeneous clinical data. To combat this, digital twins, which are virtual representations integrating clinical, multi-omic, and real-world data, enable in silico clinical trials and cohort-level simulations [213,214], offering a pathway to match AI-discovered drugs with the right patient subpopulations.

### 4.7. Regulatory Acceptance: Explainability and the Path to Market

The final bottleneck shifts from technical feasibility to regulatory oversight [215]. The “black-box” nature of deep learning hinders regulatory acceptance, as models may exploit spurious correlations (the “Clever Hans” effect), basing predictions on irrelevant artifacts rather than true biology [216,217]. Consequently, Explainable AI (XAI) techniques, such as atom-level attention in XPert and gated cross-attention in LoF-DTI [218,219], are crucial for enhancing transparency, trust, and regulatory compliance [217].

Regulatory frameworks are evolving to meet these challenges: the FDA emphasizes a flexible, risk-based credibility framework with early sponsor engagement [220,221], while the EMA proposes a structured risk-tiered approach [222]. As AI blurs the line between drug development and clinical practice (e.g., tunable personalized cell therapies) [223], agencies must evaluate both the product and the AI process itself, demanding completely new regulatory frameworks to finally bring purely AI-designed drugs to the market.

## 5. The Future Direction of AI in Drug Discovery

### 5.1. Expanding Beyond Small Molecules: ADCs and Therapeutic Peptides

Most AI drug discovery has focused on small molecules, but the next wave will address more complex modalities. Antibody–drug conjugates (ADCs) combine antibody specificity with cytotoxic payloads. This multi-component design challenge is perfectly suited for AI. Future frameworks will integrate target identification, antibody engineering, linker-payload optimization, and clinical response prediction into unified generative platforms [224]. Similarly, therapeutic peptides will be revolutionized by discrete generative models that enable controllable, Pareto-efficient optimization of sequence for binding, developability, specificity, and cellular context, unlocking previously undruggable targets [225].

### 5.2. Multimodal AI, Digital Twins, and Hybrid Mechanistic Models: From Molecules to Patients

The next frontier involves multimodal AI systems that seamlessly integrate diverse data types, including transcriptomic, epigenomic, proteomic, and spatial imaging data, along with clinical information, to construct holistic representations of disease biology. By capturing emergent properties invisible to any single modality, these models will enable more accurate predictions of therapeutic efficacy and toxicity [226].

Looking ahead, the convergence of multimodal AI with digital twins, defined as virtual patient representations built from multi-omic and real-world data, will enable in silico clinical trials, where thousands of drugs can be computationally tested before human exposure, directly addressing patient heterogeneity and reducing late-stage failures [227]. This vision aligns with the recently proposed programmable virtual human (PVH) paradigm, which extends digital twins to early drug discovery by integrating multi-scale molecular and physiological data to simulate the fate of unseen compounds directly in virtual patients, thereby enabling physiologically based drug design from the outset [228].

To ensure these simulations remain physically plausible and generalize beyond training data, a crucial evolution will be the adoption of hybrid mechanistic–AI models, which embed fundamental physical principles (e.g., molecular dynamics, pharmacokinetics) directly into neural network architectures. By combining the extrapolation power of mechanistic models with the pattern recognition capabilities of AI, these hybrid approaches promise to predict drug behavior in unseen scenarios and reduce the data hunger of purely data-driven methods [229].

### 5.3. AI-Driven Automation: Closing the Design–Make–Test Loop

Perhaps the most transformative direction is the integration of generative AI with automated high-throughput experimentation and biofoundries. These closed-loop systems combine AI-driven hypothesis generation with robotic synthesis and testing, enabling continuous cycles of learning without human intervention. This “lights-out” laboratory paradigm promises to collapse the Design–Make–Test–Analyze cycle from months to days, fundamentally accelerating the iterative optimization that drives drug discovery [230].

## 6. Conclusions and Discussion

The review is an in-depth analysis of the transformations brought by artificial intelligence in the drug discovery pipeline, including drug–target interaction prediction, protein structure prediction, de novo design, virtual screening, drug repurposing, and ADMET prediction. Graph neural networks, transformers, diffusion models, and large language models are types of AI architectures that can have a substantial impact on how drugs are discovered, making it less of a trial-and-error process and more of a data-driven approach with predictive capabilities.

Although such measures have been taken, there remains a significant gap between virtual and real life: the difference between computational prediction and clinical implementation. It must be considered that prior to enabling AI-discovered candidates to reach patients regularly, challenges such as constraints on the quality of the data, model explainability, and heterogeneity of patients, as well as changes in regulatory frameworks, need to be overcome (as explained in Section 4).

To achieve translational unity in the future, as suggested in Section 5, multimodal AI, digital twins, hybrid mechanistic systematization, and closed-loop automation with biofoundries are intended to narrow the gap between translational divides. These new paradigms will allow greater holism with respect to the representation of disease biology, personalized drug simulation, and shortening the design–make–test cycle.

For readers seeking deeper insights into specific AI methodologies or therapeutic areas, several recent reviews provide valuable perspectives. Ozdemir et al. provide a comprehensive overview of deep generative models, including diffusion models and language model-based transformers, in the field of de novo drug design [231]. Abbasi et al. provide a systematic analysis of molecular representations and generative architectures, presenting a unified framework for understanding the interplay between representation choices and model performance [232]. A critical assessment by Shree Harini and Ezhilarasan examines whether AI has truly reshaped drug discovery, noting that despite significant acceleration in early stages, no AI-only originated drug has yet achieved full regulatory approval [188].

The final test of the efficacy of AI in drug discovery is not in its algorithmic complexity but in its practical contribution to providing safer and more efficient treatment to patients by long-range collaboration between the sectors of computational, experimental, and clinical medicine.

## Figures and Tables

**Table 2:** Summary of representative protein structure prediction models.

Model	Underlying Model/Architecture	Authors & Publication Year	Main Applications (Guidance for Clinical/Drug Discovery)	Advantages/Innovations	Limitations	Validation Type	Dataset(s)	Experimental Validation	Translational Stage
RoseTTAFold All-Atom (RFAA)	RF2 extension Atomic graph for small molecules/metals/modifications RFdiffusionAA for design	David Baker et al., 2024 [64]	3D structure prediction of protein–small molecule/nucleic acid/metal/modification complexes De novo binder/sensor design	Unified single-network modeling Outperforms AutoDock Vina 1.2.0 in CAMEO (32% vs. 8%) RFdiffusionAA generates novel binders (digoxigenin, heme, bilin) Protein-only accuracy = AF2	Lower accuracy for non-protein components Ligand in correct pocket but wrong orientation Performance depends on training data	Blind CAMEO (weekly time-split) Test: PDB post May 2020	PDB: protein–small molecule (121,800/5662 clusters), protein–metal (112,546/5324), covalent (12,689/1099) +CSD small-molecule crystals +protein monomers/complexes/nucleic acids	Prediction: None (computational only) Design: Yes—binding/crystal for digoxigenin (Kd = 343 nM), heme (0.86 Å RMSD), bilin	Preclinical (in silico + in vitro)
NeuralPLexer	Multi-scale deep generative model (CPM + ESDM) Molecular Heat Transformer (MHT) for graph encoding	Zhuoran Qiao et al., 2024 [65]	Protein-ligand complex structure prediction Structure-based drug design (e.g., KRAS, GPCRs)	Predicts ligand-induced conformational changes Outperforms existing methods in blind docking & binding site recovery Samples both apo and holo states Equivariant diffusion with chirality constraints	Largely limited to crystal-like structures, not physiological conformations. Not applicable to post-translational modifications or large multi-state complexes. Limited for protein families without experimentally determined homologs.	External (time split on PDBbind2020; sequence split on PocketMiner)	PL2019-74k, PDBbind2020, PocketMiner, recent PDB (118 complexes)	None (computational study only)	Preclinical (in silico discovery)
DynamicBind	An E(3)-equivariant, diffusion-based, graph neural network utilizing a coarse-grained representation.	Wei Lu et al., 2024 [66]	Undruggable targets Cryptic pockets Global docking Virtual screening	Large-scale conformational changes (e.g., DFG-in/out) No holo-structure needed Predicts cryptic pockets >MD simulation efficiency SOTA docking (33% success) & screening (auROC 0.68)	Limited generalization to proteins with low sequence homology	External Time-split (pre-2019 train) Independent test (2020+)	PDBbind2020 MDT (599) Antibiotics (2616 pairs)	None (computational only)	Preclinical (in silico)
Umol (Universal molecular framework)	A neural network extended from AlphaFold2’s EvoFormer architecture.	Patrick Bryant et al., 2024 [67]	Predict protein-ligand complex from sequence + SMILES (no native structure needed). Early drug discovery & repurposing for targets without holo-structure.	No protein structure required. pIDDT confidence metric predicts accuracy & affinity. Umol-pocket achieves 45% success rate (PoseBusters). 98% ligand validity (RDKit).	Without pocket: success rate drops to 18% (overfitting). Requires MSA. Raw predictions may have clashes (relaxation needed). Computational: GPU, >1000 residues may need cropping.	external on PoseBusters (structures not in training set, <30% seq identity)	Training set: 16,420 protein-ligand complexes from PDBbind 2019, clustered at 20% sequence identity.	None (computational only)	Preclinical (in silico)
FABind	E(3)-equivariant Graph Neural Network (GNN) comprising a series of geometry-aware update layers.	Qizhi Pei et al., 2023 [68]	Early-stage drug discovery Targets with unresolved or unknown binding pockets	Fast: >170× faster than DiffDock End-to-end: no external modules (e.g., P2Rank) needed Accurate: scheduled sampling + distance map loss + iterative refinement Generalizable: strong performance on unseen proteins	Protein modeled at residue level (assumes rigidity) Cannot handle multiple binding sites or multiple ligand conformations	Time-based split (pre-2019 train/val, post-2019 test) Additional unseen protein evaluation (UniProt ID filter)	PDBbind v2020	None (computational only)	Preclinical (in silico)
tFold System	ESM-PPI + Evoformer-Single + SE(3) module	Wu F., Zhao Y., Wu J. et al., 2026 [69]	De novo epitope-specific antibody design Ab/Ag structure prediction Virtual screening	MSA-free → 50× faster than AF3 High accuracy (CDR-H3 RMSD 3.01 Å) Enables epitope targeting Validated nM-pM affinities	Limited training data → lower on nanobody complexes	Time-based split (pre-2022 train, 2022 test)	SAbDab, Thera-SAbDab, OAS, CoV-AbDab	Yes (SPR, competition assays, phage display)	Preclinical (in silico + in vitro)

**Table 5:** Summary of representative AI models for ADMET property prediction.

Model	Underlying Model/Architecture	Authors & Publication Year	Main Applications (Guidance for Clinical/Drug Discovery)	Advantages/Innovations	Limitations	Validation Type	Dataset(s)	Experimental Validation	Translational Stage
ADMETlab 2.0 Integrated Prediction Model (covers 88 endpoints)	Multi-task Graph Attention (MGA) with RGCN, attention, and fully connected layers	Guoli Xiong et al., 2021 [158]	Early drug discovery, lead optimization, rapid in silico prescreening	Predicts 88 endpoints (physicochemical, medchem, ADME, toxicity) First MGA platform for classification & regression Batch: ~84 s/1000 molecules No descriptor calculation	Low AUC for some CYPs (CYP1A2 substrate 0.737; CYP2C9 substrate 0.725) Low R^2^ for Clearance (0.678) and LC50 DM (0.524)	Original: Internal test set (separate) Supplement: Computational prediction + in vitro CYP inhibition assay (HLMs) [159]	ChEMBL, PubChem, OCHEM, Tox21, the literature, proprietary	Original: None (computational only) Supplement: Yes—in vitro CYP inhibition; IC50: CYP1A2 230 µg/mL, CYP2C8 213 µg/ML [159]	Original: Preclinical (in silico) Supplement: Preclinical (HDI screening) [159]
FP-GNN (Fingerprints and Graph Neural Networks)	GAT + ANN; integrates molecular graphs with MACCS, PubChem, ErG fingerprints	Hanxuan Cai et al. 2022 [160]	Molecular property prediction (physicochemical, bioactivity, ADMET)	Combining graphs with multiple fingerprints Outperforms SOTA on 13 public, LIT-PCBA, and 14 breast cell line datasets Strong anti-noise ability Interpretable (identifies key fragments)	Performance depends on fingerprint choice (mixed > Morgan) Optimal graph/fingerprint balance varies per dataset	Random (8:1:1), scaffold-based, asymmetric embedding (3:1)	13 public benchmarks; LIT-PCBA (15 targets); 14 breast cell lines	Original: None (computational only) Supplement: Yes CDK9 kinase inhibition (IC50 214–504 nM) Anti-proliferation on 8 cancer cell lines (e.g., HeLa, MOLM-13) Western blot, apoptosis, stability assays [161]	Original: Preclinical (in silico) Supplement: Preclinical (in silico + in vitro validation) [161]
MoLFormer-XL (Molecular Language Transformer)	Transformer encoder with linear attention + rotary embeddings; pre-trained as MLM on SMILES	Jerret Ross et al., 2022 [162]	Virtual screening; predicts quantum to physiological properties	Pre-trained on > 1B SMILES Linear attention reduces GPUs ~1000→16 SOTA on many MoleculeNet tasks Captures spatial interatomic relations	Worse than 3D GNNs (e.g., SchNet) on quantum geometry tasks Performance depends on pre-training data scale/diversity No experimental validation	Internal: scaffold splits (classification), random splits (regression); 5-fold CV for QM9	Pre-training: PubChem (111M) + ZINC (>1B) Fine-tuning: MoleculeNet (BBBP, Tox21, QM9, ESOL, etc.)	None (computational only)	Preclinical (in silico)
GEM(Geometry-enhanced molecular representation learning method)	GeoGNN: dual-graph (atom-bond + bond-angle) Geometry SSL: bond length, angle, distance matrix prediction	Xiaomin Fang et al., 2022 [163]	Predict quantum & physicochemical properties	SOTA on 14/15 MoleculeNet benchmarks Explicit geometry encoding Pre-trained on 20 M molecules	Cannot effectively represent dihedral angles. Lacks electronic structure information (e.g., atomic charges, bond orders) [164].	Pre-train: random split Downstream: scaffold split	Pre-train: ZINC15 (20 M) Downstream: 15 MoleculeNet tasks	None (in silico only)	Preclinical (in silico)
ADMET-AI	Chemprop-RDKit (GNN + 200 RDKit features)	Kyle Swanson et al., 2024 [165]	Early-stage drug discovery High-throughput docking Filtering generative AI compound libraries Prioritizing candidates with optimal ADMET profiles	Highest avg. rank on TDC ADMET Leaderboard Fastest public ADMET web server (45% faster) DrugBank reference with ATC filter Local batch: 1 M molecules in 3.1 h (GPU) Open-source	None explicitly mentioned	Internal (5 predefined train/val/test splits per dataset)	41 TDC ADMET datasets (10 regression, 31 classification)	None (computational only)	Preclinical (in silico)
Therapeutics Data Commons (TDC)	Platform of AI-ready datasets, tasks, benchmarks; three-level hierarchy	Kexin Huang et al., 2022 [166]	Evaluate AI for lead optimization, virtual screening, generative design; guide method selection	66 AI-ready datasets (15.9 M points) Multiple data splits (scaffold, temporal, cold-start) 17 generation oracles Public leaderboards TDC open-source Python package (website: http://tdcommons.ai, accessed 20 April 2026)	TDC reports higher clearance values for shared molecules [167]. Combining datasets introduces inconsistencies, compromising ML model quality [167].	Benchmark-based; splits: scaffold, temporal, cold-start, combination	66 datasets: small molecules (15 tasks), macromolecules (8 tasks), cell/gene therapies (2 tasks)	None (computational platform only)	Preclinical (in silico)—target discovery, activity, efficacy/safety, manufacturing
OmniMol	Multi-task framework with task-routed MoE SE(3)-equivariant graph encoder Hypergraph view	Bowen Wang et al., 2025 [168]	Predict ADMET properties SAR studies Drug candidate screening/optimization	Handles imperfect labels (O(1) complexity) Explainable (molecule, property, molecule-property relations) Chirality-aware (SE(3) encoder) Learning-based conformational relaxation	Small drug-like molecules only No continuous prediction for binary tasks Retrospective; needs prospective validation Some proprietary data	Internal 8:1:1 random split External on chirality/SAR	ADMETlab 2.0 (40 class, 12 regress) PubChem enantiomer pairs Chiral cliff dataset	None (computational only)	Preclinical (in silico)

**Table 6:** Representative AI models integrated into clinical workflows to facilitate translation.

Model	Underlying Model/Architecture	Authors & Publication Year	Main Applications (Guidance for Clinical/Drug Discovery)	Advantages/Innovations	Limitations	Validation Type	Dataset(s)	Experimental Validation	Translational Stage
TxGNN	Uses a Graph Neural Network (GNN) encoder and a metric learning module, pre-trained on a large-scale medical knowledge graph.	Huang, K., Chandak, P., et al. (2024) [178]	Drug repurposing: Identifies candidate drugs for diseases with no existing treatments or limited molecular data, providing ranked lists of therapeutic candidates to guide clinical investigation.	Zero-shot prediction for diseases with no drugs Outperforms 8 baselines (up to +49.2% AUPRC) Multi-hop interpretable explanations	Performance depends on KG quality, completeness, and biases Small pilot human evaluation (n = 12) Potential unmeasured confounders in EMR analysis	Zero-shot evaluation on held-out diseases; EMR validation (1.27 M patients)	Custom KG (17,080 diseases, 7957 drugs); Mount Sinai EMRs	None (computational/retrospective only)	Preclinical (in silico)
HEX (H&E to protein expression)	Pathology foundation model (e.g., MUSK) + Feature Distribution Smoothing (FDS) + Adaptive Loss Function (ALF)	Li Z, et al. (2026) [179]	Prognosis prediction (early-stage NSCLC) ICI response prediction (advanced NSCLC)	Virtual spatial proteomics from H&E Low-cost, scalable MICA improves prognosis by 22%, ICI response by 24–39%	Not explicitly reported in the paper	Cross-validation Independent external	Train: Stanford-WSI (10 WSIs) Val: Stanford-TMA, TA-TMA External: Bern (34 tissue types) Clinical: NLST, TCGA, PLCO (n = 2150); Stanford-IO (n = 148)	IHC validation (CD31, Ki-67)	Retrospective clinical + IHC validation
DeepRare	Multi-agent system powered by LLM (DeepSeek-V3) with MCP-like architecture, integrating 40+ specialized tools and knowledge sources.	Zhao W, Wu C, Fan Y, et al. (2026) [180]	Rare disease differential diagnosis. Input: free-text, HPO, VCF. Output: ranked diagnoses + traceable reasoning.	Recall@1 57.18% (HPO), +23.79% vs. next best. Multi-modal (HPO + gene) 69.1% vs. Exomiser 55.9%. Traceable reasoning, 95.4% expert agreement. Outperforms expert physicians (64.4% vs. 54.6%).	Reasoning weighing error (41% failures). Phenotypic mimic diagnosis (38.5%). No built-in screening for non-specialists	Internal & external; vs. 15 methods & physicians; ablation.	9 datasets, 6401 cases, 2919 diseases (RareBench, MyGene2, DDD, MIMIC-IV-Rare, Xinhua, Hunan).	None (computational). Expert review of reasoning (95.4% agreement).	Clinical validation; web app deployed as copilot.
AMIE (Articulate Medical Intelligence Explorer)	LLM-based system built on Gemini 2.0 Flash with multi-step inference (web search + self-critique)	O’Sullivan JW, Palepu A, et al. (2026) [181]	Assisting general cardiologists in triage, diagnosis, and management of patients with suspected genetic cardiomyopathies using real-world clinical data.	Reduces errors and omissions vs. cardiologists alone Saves clinician time (50.5% of cases) Open-sourced real-world dataset	Limited to text-based reports (not raw images) Single-center US data, no history/physical exam 6.5% clinically significant hallucinations	Randomized Controlled Trial (RCT), blinded subspecialist evaluation	107 real-world patients from Stanford Center for Inherited Cardiovascular Disease (SCIDC)	RCT with 9 cardiologists, 107 cases (retrospective data)	Clinical research (RCT completed, awaiting prospective deployment)
ccRCC Multimodal AI Predictor	ICI: CatBoost (gradient boosting) TKI: Logistic regression Features: transcriptomic signatures	Stupichev D, Mihecheva N, Postovalova E, et al. (2025) [182]	Predict ICI & TKI response in ccRCC Guide first-line therapy selection (ICI vs. TKI)	Largest harmonized ccRCC transcriptomic DB (n = 3621) 5 HiTME subtypes validated by MxIF Outperforms existing biomarkers	No data for lenvatinib + pembrolizumab, etc. No prospective validation Small subgroups Normalization may mask batch biology	Retrospective, multi-cohort independent validation	Train: IMmotion151,150, CheckMate, E-MTAB-3267 Valid: JAVELIN, WU-RCC, COMPARZ, etc.	Partial (HiTME by MxIF, n = 34); prediction models computational only	Retrospective validation; framework for clinical use proposed
Brainomix 360 Stroke Imaging Software	AI imaging algorithms for CT analysis (including e-ASPECTS, e-CTA, e-CTP modules)	Nagaratnam K, Neuhaus AA, et al. (2025) [183]	Guidance for clinical decision-making in acute stroke triage and treatment	Increases thrombectomy rates Automated detection/quantification Real-time image sharing	Not reported in this study	Prospective observational (pre/post + patient-level)	SSNAP (England, n = 452,952, 107 hospitals)	Clinical study only	Clinical (real-world implementation)
Autonomous clinical AI agent	GPT-4 + tool calling + RAG + vision transformers (MSI/KRAS/BRAF) + MedSAM + OncoKB + PubMed/Google search	Ferber D, et al. (2025) [184]	Guidance for personalized treatment decisions in oncology	Autonomously selects and uses multiple medical tools to solve complex multimodal patient cases	Small sample size (n = 20) Cloud-based GPT-4 (privacy/regulatory) Single-slice radiology only	Internal, blinded, 4 experts	20 simulated multimodal cases; TCGA; in-house/public CT/MRI; ~6800 guidelines	None (computational)	Preclinical (proof-of-concept)
CONCH	CoCa (ViT-B/16 + GPT text encoder + multimodal decoder)	Lu MY, Chen B, Williamson DFK, et al. (2024) [185]	Zero-shot classification (tile/WSI) Cross-modal retrieval (i2t/t2i) Zero-shot segmentation Weakly supervised WSI classification	Largest histopathology pretraining (1.17 M pairs) SOTA on 14 benchmarks Open-source	Small pretraining scale vs. billion-scale models Poor zero-shot on rare diseases/many classes Potential train–test data overlap	Internal validation on public benchmarks & held-out sources	Pretrain: 1.17 M pairs (PubMed, education) Test: TCGA, DHMC LUAD, CRC100k, SICAP, DigestPath, EBRAINS, etc.	None (computational only)	Preclinical (in silico)
AMIE	Large language model (based on PaLM 2) with instruction fine-tuning and self-play simulated dialogue environment	Tu T, et al. (2025) [186]	Diagnostic dialogue & history-taking in primary care	Higher diagnostic accuracy than PCPs (RCT) Superior on 30/32 specialist axes & 25/26 patient-actor axes	Text-chat interface unfamiliar; not real-world practice Most scenarios assumed disease state Fairness/bias issues	Randomized double-blind crossover (remote OSCE)	Real: MedQA, MultiMedQA, MIMIC-III, 98,919 dialogues Simulated: 11,686 per iteration (5230 conditions) Eval: 159 OSCE cases	None (computational)	Clinical research (simulated OSCE)
Self-supervised learning strategy for postoperative brain cavity segmentation	3D CNN (U-Net variant) Resection simulation (geodesic polyhedron + noise, CSF blending, anatomical constraints)	Perez-Garcia, F. et al., (2021) [187]	Segment resection cavities on postoperative T1w MRI Quantify resected structures for epilepsy surgery	Self-supervised training from unlabeled preoperative MRI—no large annotated data needed Realistic cavity shape, CSF filling, anatomical constraints	Poor performance for very small cavities or large brain shift/edema Cuboid-shaped simulation underperforms	Internal (EPISURG) External (Milan, Paris, Strasbourg) Qualitative (BITE, intraop MRI)	Pre-training: IXI, ADNI, OASIS (1813 unlabeled) Fine-tune/eval: EPISURG, Milan, Paris, Strasbourg (20–133 annotated) BITE (qualitative)	None (computational only)	Preclinical (in silico)

## Data Availability

No new data were created or analyzed in this study. Data sharing is not applicable to this article.

## Abbreviations

Abbreviation	Full Form
AARD%	Average Absolute Relative Deviation percent
AD	Alzheimer's Disease
ADC	Antibody-Drug Conjugate
ADMET	Absorption, Distribution, Metabolism, Excretion, and Toxicity
ADMET-AI	Absorption, Distribution, Metabolism, Excretion, and Toxicity-Artificial Intelligence
ADMETlab	Absorption, Distribution, Metabolism, Excretion, and Toxicity Laboratory
AF2	AlphaFold 2
AF3	AlphaFold 3
AI	Artificial Intelligence
AKT1	AKT serine/threonine kinase 1
AL	Active Learning
AR-SBDD	Autoregressive Structure-Based Drug Design
AUC	Area Under the Curve
AUPRC	Area Under the Precision-Recall Curve
AutoML	Automated Machine Learning
BAN	Bilinear Attention Network
BBB	Blood-Brain Barrier
BGMM	Bayesian Gaussian Mixture Model
CDAN	Conditional Domain Adversarial Network
CDK9	Cyclin-Dependent Kinase 9
CDR	Complementarity-Determining Region
CNN	Convolutional Neural Network
CNS	Central Nervous System
CReM	Chemically Reasonable Mutations
CRISP	Cell-Type-Specific Drug Perturbation Responses Predictor
CSA	Coupled Simulated Annealing
CTRP	Cancer Therapeutics Response Portal
CVAE	Conditional Variational Autoencoder
DDPM	Denoising Diffusion Probabilistic Model
DeepSP	Deep Learning for Spatial Properties
DFT	Density Functional Theory
DHMC	Dartmouth-Hitchcock Medical Center
DL	Deep Learning
DMTA	Design-Make-Test-Analyze
D-MPNN	Directed Message Passing Neural Network
DrugBAN	Drug Bilinear Attention Network
DrugMAN	Drug Mutual Attention Network
DSN	Domain Separation Network
DTA	Drug-Target Affinity
DTI	Drug-Target Interaction
EC	Enzyme Commission
EGFR	Epidermal Growth Factor Receptor
EGNN	E(3)-Equivariant Graph Neural Network
EHR	Electronic Health Record
ELISA	Enzyme-Linked Immunosorbent Assay
EMA	European Medicines Agency
EMR	Electronic Medical Record
ESMFold	Evolutionary Scale Modeling Fold
FABind	Fast and Accurate Binding
FDA	Food and Drug Administration
FDTIIT	Flexible drug-target interaction prediction with interactive information extraction and trade-off
FFN	Feed-Forward Network
FP-GNN	Fingerprints and Graph Neural Networks
FXS	Fragile X Syndrome
GAN	Generative Adversarial Network
GAT	Graph Attention Network
GCN	Graph Convolutional Network
GEM	Geometry-enhanced molecular representation learning
GEO	Gene Expression Omnibus
GeoGNN	Geometry-enhanced Graph Neural Network
GNN	Graph Neural Network
GPCR	G Protein-Coupled Receptor
GPT	Generative Pre-trained Transformer
HPO	Human Phenotype Ontology
IC50	Half maximal inhibitory concentration
IgFold	Immunoglobulin Fold
IPA	Invariant Point Attention
KG	Knowledge Graph
KGE	Knowledge Graph Embedding
LaF	Labels as Features
LBVS	Ligand-Based Virtual Screening
LDHA	Lactate Dehydrogenase A
Lib-INVENT	Library-INVENT
LINCS	Library of Integrated Network-based Cellular Signatures
LLM	Large Language Model
LM	Language Model
LSSVM-AC	Least Squares Support Vector Machine with Atom Contribution
LSTM	Long Short-Term Memory
LUAD	Lung Adenocarcinoma
mAbs	monoclonal antibodies
MCC	Matthews Correlation Coefficient
MD	Molecular Dynamics
MDP	Masked Drug Patch
MFGP	Masked Functional Group Prediction
MGA	Multi-task Graph Attention
MHT	Molecular Heat Transformer
ML	Machine Learning
MLM	Masked Language Model
MLPANN-AC	Multilayer Perceptron Artificial Neural Network with Atom Contributio
MMP	Matched Molecular Pair
MoA	Mechanism of Action
MoE	Mixture of Experts
MoLFormer-XL	Molecular Language Transformer - Extra Large
MolTrans	Molecular Interaction Transformer
Mpro	Main protease
MSA	Multiple Sequence Alignment
NeuralPLexer	Neural Protein-Ligand Complex Structure Predictor
NFM	Neural Factorization Machine
NLP	Natural Language Processing
NMR	Nuclear Magnetic Resonance
OEKRF	Ontology-based Knowledge Retrieval Framework
OOD	Out-Of-Distribution
OPTICS	Ordering Points To Identify the Clustering Structure
PBCT	Predictive Bi-Clustering Trees
PDB	Protein Data Bank
pIDDT	predicted interatomic distance difference test
PLM	Protein Language Model
PMDM	Pocket-based Molecular Diffusion Model
PPI	Protein-Protein Interaction
pre-LN	Pre-Layer Normalization
PROTACs	Proteolysis-Targeting Chimeras
PTM	Post-Translational Modification
PVH	Programmable Virtual Human
QED	Quantitative Estimate of Drug-likeness
QSAR	Quantitative Structure-Activity Relationship
RBF	Radial Basis Function
RBD	Receptor-Binding Domain
RCT	Randomized Controlled Trial
RELATION	REceptor-LigAnd interacTION
RFAA	RoseTTAFold All-Atom
RGCN	Relational Graph Convolutional Network
RL	Reinforcement Learning
RMSD	Root-Mean-Square Deviation
RNN	Recurrent Neural Network
RWD	Real-World Data
RWR	Random Walk with Restart
SAR	Structure-Activity Relationship
SBDD	Structure-Based Drug Design
SBVS	Structure-Based Virtual Screening
SCScore	Synthetic Complexity Score
SEM	Synthesizability Estimation Model
SHGCL-DTI	Semi-supervised Heterogeneous Graph Contrastive Learning for Drug–Target Interaction prediction
SMILES	Simplified Molecular-Input Line-Entry System
SOTA	State-Of-The-Art
SPR	Surface Plasmon Resonance
SS-HPBCT	Semi-Supervised Hybrid Predictive Bi-Clustering Trees
SSL	Self-Supervised Learning
STAR	Structure–Tissue Exposure/Selectivity–Activity Relationship
TCGA	The Cancer Genome Atlas
TDC	Therapeutics Data Commons
TNIK	Traf2- and Nck-interacting kinase
ToxACoL	Adjoint Correlation Learning for Multi-Condition Acute Toxicity Assessment
TransformerCPI	Transformer for Compound-Protein Interaction
TRPA1	Transient Receptor Potential Ankyrin 1
TRPM8	Transient Receptor Potential Melastatin 8
TTD	Therapeutic Target Database
VAE	Variational Autoencoder
VCF	Variant Call Format
VHL	Von Hippel-Lindau
VOCs	Variants of Concern
vScreenML	virtual screening machine learning
WSI	Whole Slide Image
XAI	Explainable Artificial Intelligence
XG4Repo	eXplainable Graphs for Repurposing
